# Lesser suppression of response to bright visual stimuli and visual abnormality in children with autism spectrum disorder: a magnetoencephalographic study

**DOI:** 10.1186/s11689-019-9266-0

**Published:** 2019-06-14

**Authors:** Sho Aoki, Kuriko Kagitani-Shimono, Junko Matsuzaki, Ryuzo Hanaie, Mariko Nakanishi, Koji Tominaga, Yukie Nagai, Ikuko Mohri, Masako Taniike

**Affiliations:** 1 Division of Developmental Neuroscience, United Graduate School of Child Development, Osaka University, 2-2, Yamadaoka, Suita, Osaka, 565-0871 Japan; 2 Molecular Research Center for Children’s Mental Development, United Graduate School of Child Development, Osaka University, 2-2, Yamadaoka, Suita, Osaka, 565-0871 Japan; 3 Department of Pediatrics, Osaka University Graduate School of Medicine, Osaka, Japan; 4 National Institute of Information and Communications Technology, Osaka, Japan

**Keywords:** Autism spectrum disorders (ASD), Visual abnormality, Neural suppression, Bright visual stimuli, Supramarginal gyrus (SMG), Magnetoencephalography (MEG)

## Abstract

**Background:**

Visual abnormality is a common sensory impairment in autism spectrum disorder (ASD), which may cause behavioral problems. However, only a few studies exist on the neural features corresponding to the visual symptoms in ASD. The purpose of this study was to investigate the relationship between cortical responses to visual stimuli and visual abnormality to examine the neurophysiological mechanisms of the visual abnormality in ASD.

**Methods:**

Twenty-two high-functioning children with ASD (10.95 ± 2.01 years old) and 23 age-matched typically developing (TD) children (10.13 ± 2.80 years old) participated in this study. We measured the cortical responses (i.e., activated intensity and attenuation ratio) elicited by the Original visual image and other two types of bright images (the Dot noise or Blind image, which includes overlapped particles onto the Original image or the enhanced-brightness version of the Original image, respectively) using magnetoencephalography.

**Results:**

The severity of visual abnormalities was significantly associated with behavioral problems in children with ASD. In addition, we found the increased cortical activation in response to the Original image in the left supramarginal gyrus (SMG) and middle temporal gyrus in children with ASD. However, there were no inter-group differences in the primary visual and medial orbitofrontal cortices. Furthermore, when we compared cortical responses according to the type of images, children with ASD showed lesser attenuation of the activated intensities than children with TD in response to the bright images compared with the Original image in the right SMG. These attenuation ratios (Dot noise/Original and Blind/Original) were also associated with the severity of visual abnormalities.

**Conclusions:**

Our results show that dysfunction of stimulus-driven neural suppression plays a crucial role in the neural mechanism of visual abnormality in children with ASD. To the best of our knowledge, this is the first magnetoencephalography study to demonstrate the association between the severity of visual abnormality and lower attenuation ratios in children with ASD. Our results contribute to the knowledge of the mechanisms underlying visual abnormality in children with ASD, and may therefore lead to more effective diagnosis and earlier intervention.

**Electronic supplementary material:**

The online version of this article (10.1186/s11689-019-9266-0) contains supplementary material, which is available to authorized users.

## Background

Autism spectrum disorder (ASD) is a neurodevelopmental disorder that affects social interaction and behavioral flexibility [1]. Sensory abnormalities are also core features of ASD, and have been reported to show a high prevalence in previous studies [2–4]. In addition, sensory abnormalities interrupt behavioral adaptation and cause behavioral problems in individual with ASD [3, 5]. Since the neural bases underlying these abnormalities have recently been receiving considerable interest from the aspect of leading to more effective diagnosis and intervention, it is important to examine the neural features corresponding to each symptom [6, 7].

The number of visual sensory studies in ASD, as well as that of other sensory modalities, has been increasing in recent years [8]. Adults with ASD were reported to experience several specific patterns of visual sensory abnormality in a preliminary study [9]. However, the underlying mechanism has not been elucidated yet.

Visual sensory abnormality in ASD can be considered in the following framework. First, sensory symptoms in ASD may be caused by abnormality in the basic visual processing, i.e., patients with ASD exhibiting sensory symptoms experience sensory stimuli more intensely in the primary visual cortex. This is derived from the idea that enhanced low-level visual perception, which has been replicated [10, 11], could itself lead to visual sensory abnormality in ASD [12]. Further, in recent years, a few studies have revealed the relationship between the atypical response amplitude to low-level stimuli and sensory symptoms in children with ASD [13, 14]. Although these findings suggest that the visual sensory abnormality can be associated with the early stage of visual processing at the occipital sites, no study has yet revealed whether higher order processing is affected in children with ASD.

The second possibility is associated with the emotional pathways, i.e., patients with ASD exhibiting sensory abnormality fundamentally experience/process sensory stimuli in a manner identical to that of healthy individuals, but have a different emotional or behavioral reaction. This is the idea of late stage abnormality. Recently, the response amplitude to auditory stimuli in the late stage has been shown to be related to emotional problems in adolescents with ASD [15]. Abnormal responses to emotional stimuli have also been demonstrated in the orbitofrontal cortex of youth with ASD [16, 17].

In addition, an external sensory event such as a sudden noise or a flash of light draws our attention. This is called stimulus-driven attention [18]. Atypical patterns of stimulus-driven attention has been observed in the late stage [19, 20], and which have been shown to exhibit abnormal activation in the inferior parietal region among patients with ASD [21, 22]. Therefore, although visual sensory abnormality is suggested to be associated with the brain areas responsible for emotional regulation and reorienting attention, no study has yet reported a direct relationship between the activation of these areas in response to visual stimuli and the visual sensory symptoms of ASD.

Finally, the third possibility refers to abnormality in habituation or repetition suppression, which decreases the neural responses following stimulus repetition [23]. Sensory habituation in response to repeated sounds has been shown to be impaired in infants at high risk for ASD and patients with ASD [24–26]; moreover, this phenomenon might be involved in the neural basis of visual sensory abnormality in ASD.

Magnetoencephalography (MEG) is an electrophysiological modality involving direct and noninvasive access, with temporal resolution in the range of milliseconds and high spatial resolution [27]. The previous studies which have examined the neural mechanism of sensory abnormality using MEG have mainly focused on auditory symptoms [26, 28, 29], and there are no previous studies examining visual sensory abnormality in ASD.

The aim of this study was using MEG to examine the neural mechanisms underlying visual sensory abnormality in children with ASD. The current study also prepared different textual stimuli overlapped onto object pictures, and investigated the direct relationship between visual evoked responses and sensory symptoms. This approach may allow reproduction of the abnormal visual input which children with ASD exhibiting sensory abnormality experience in their real environment, and it helps us understand the underlying physiological mechanisms based on the symptoms. We hypothesized that not only the primary sensory cortex but also stimulus-driven attention and emotional regulation could contribute to the visual sensory abnormality in ASD. Furthermore, these abnormal responses could be associated with the severity of visual sensitivity in ASD.

## Methods

### Participants

We recruited 31 high-functioning children with ASD and 28 age-matched typically developing (TD) children in this study. The children with ASD were diagnosed at the Department of Pediatrics in the Osaka University Hospital or another hospital in Osaka Prefecture. The diagnosis was confirmed by experienced developmental pediatricians based on the Diagnostic and Statistical Manual of Mental Disorders, Fifth Edition criteria (DSM-5) [1] using the information from the Autism Diagnostic Observation Schedule, Generic [30] or Second Edition [31, 32], obtained by licensed psychologists, and the parent-reported Japanese version of the Social Communication Questionnaire (SCQ) [33, 34]. One patient with epileptic discharges was excluded from this study. At the time of testing, eight participants with ASD were receiving medication: methylphenidate in five cases, atomoxetine in one, selective serotonin reuptake inhibitors in one, and atypical antipsychotic medication in two.

The TD children were recruited through a public newsletter distributed in Osaka prefecture. Children who had a history of neurological or neurodevelopmental disorders or had received special education were excluded from the TD group. In addition, to exclude autistic traits, SCQ was completed by all the TD participants. Only the participants with lower scores in the SCQ than the cut-off value were included in this study.

Cognitive functioning was assessed using the Wechsler Intelligence Scale for Children, Fourth Edition in all children. The high-functioning participants whose full-scale intelligence quotient (FSIQ) scores were more than 75 were included in this study. The characteristics of their visual abnormalities were assessed using the new Japanese version of the Sensory Profile (SP) [35, 36], in which a higher score corresponds to greater severity of sensory symptoms. Furthermore, the characteristics of behavioral problems were measured using the Japanese version of the Child Behavior Checklist (CBCL) [37, 38]. Both questionnaires were completed by the children’s guardians.

To add to the above inclusion/exclusion criteria, all children were confirmed to be right-handed using the Edinburgh Handedness Inventory [39], and had no history of genetic syndromes or apparent sensory impairment, based on their parents’ report.

The final data used for the current analyses were obtained from 22 children in the ASD group and 23 children in the TD group because of exclusion for excessive movement or noncompliance (*n* = 8); failure to meet the DSM-5 criteria of ASD or the high-functioning criteria, as characterized by FSIQ scores less than 75 (*n* = 4); epileptic discharges (*n* = 1); or equipment failure (*n* = 1).

Written informed consent for participation, in accordance with the principles of the Declaration of Helsinki, was obtained from all participants and their guardians. The study was approved by the Institutional Review Board of Osaka University Hospital. Participants received a gift card as compensation for participation.

### Visual stimuli

**Fig. 1:**
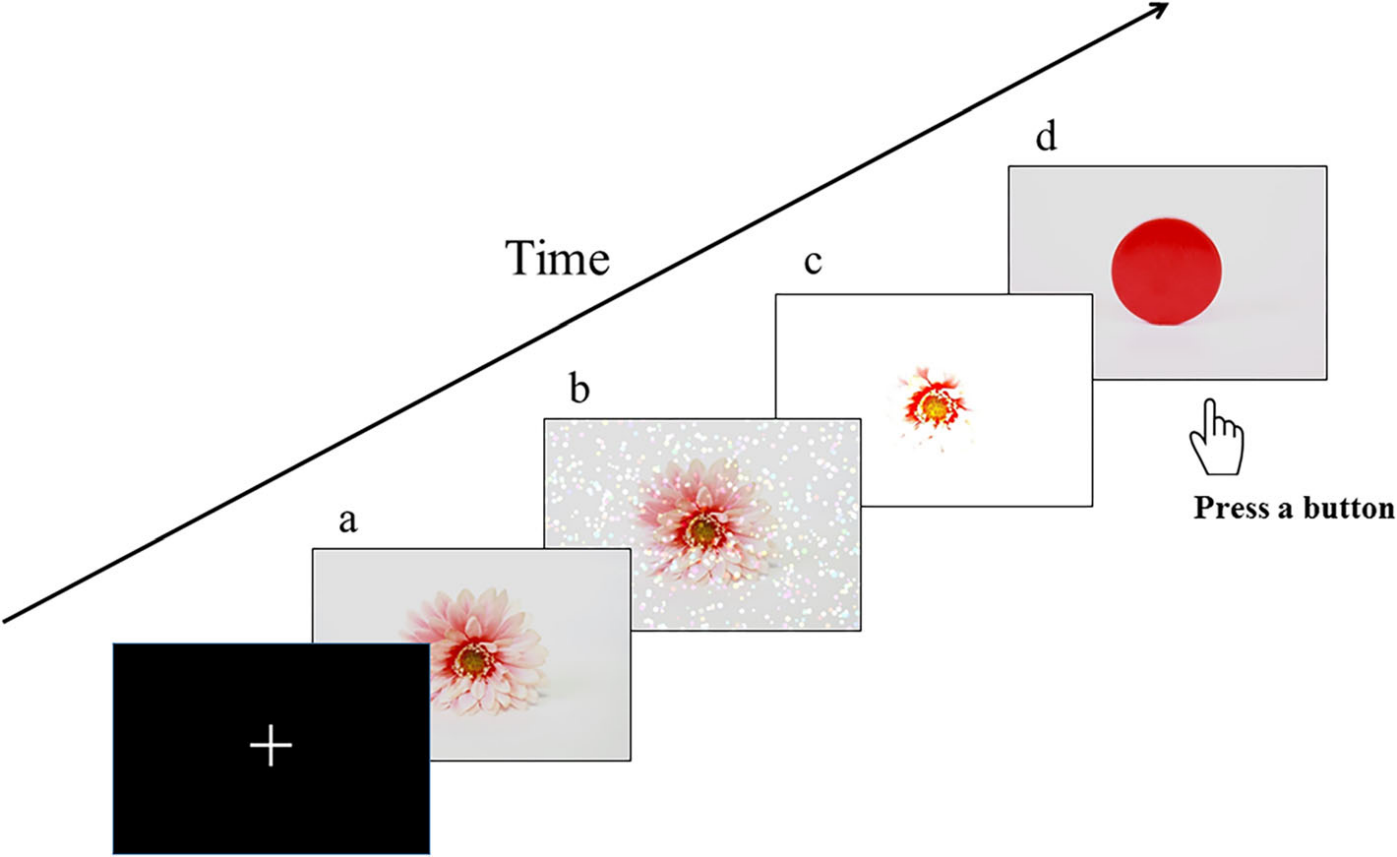
Visual stimuli and trial timeline. Visual stimuli consisted of three types of pictures. The picture **a** is the Original image. The picture **b** is the Dot noise image, which involved superimposed bright dots on the Original image. The picture **c** is the Blind image, which is obtained after enhancing the luminance of the Original image. To determine whether the participants are alert, they are instructed to press the button for the cue picture **d**

The visual stimuli used consisted of three types of images (Fig. 1): (a) the Original image, composed of color pictures of an object, such as a toy tree, excluding pictures of humans and human-like objects; (b) the Dot noise image, which consisted of superimposed bright dots on the Original image; and (c) the Blind image, which was obtained by enhancing the brightness of the Original image. These bright images, including the Dot noise and Blind images, were selected based on our pilot investigation. This investigation was administered through a questionnaire based on some printed images, which included various types of visual stimuli superimposed on the Original image [9]. Sixty-one children with ASD, as diagnosed by experienced developmental pediatricians based on DSM-5 [1], and 58 age-matched children from the community participated in this investigation. The children with ASD had higher visual item scores for SP than that of community children. They were asked to provide their answers regarding the images based on the correspondence of the images to unusual visual experiences from their life. The result showed that children with ASD more frequently reported the Dot noise image as corresponding to their unusual visual experiences (*χ*^*2*^ = 4.96; *p* < 0.05). Furthermore, although the group difference was not significant (*χ*^*2*^ = 0.24; *p* = 0.628), more than 25% of children with ASD reported the Blind image.

The average luminance, measured using QUALIX Lite (Iwasaki Electric Co., Ltd., Tokyo, Japan), was 48.14 ± 11.56, 55.83 ± 11.70, and 84.93 ± 20.39 cd/m^2^ for Original, Dot noise, and Blind images, respectively. Repeated measure analyses of variance showed a main effect of stimulus (*F*(1.250, 23.757) = 87.50, *p* < 0.001, *η*_*p*_^*2*^ = 0.82). Post-hoc comparisons revealed significant differences among all images (*p* < 0.001). Additionally, to confirm that the participants were alert, they were instructed to press a button when the cue picture (a red circle, displayed 15 times; Fig. 1d) was shown in a pseudorandom order. A fixation point was presented at the center of the screen at the beginning of the task with the instruction that each participant looked the place where the fixation point was given (Fig. 1). All images were projected onto a screen 325 mm from the participants’ eyes using a presentation software (Neurobehavioral Systems, Berkeley, CA, USA) and a liquid-crystal projector (LVP-HC6800, Mitsubishi Electric, Tokyo, Japan). Each image was subsequently presented 100 times in a pseudorandom order for 3000 ms. An inter-trial interval was not adopted in order to present the required number of each type of image within a period of time children can maintain their attention and yet to avoid artifacts such as eye blinks and body movement by switching images too fast.

### MEG and magnetic resonance imaging recordings

Before MEG recordings, the three-dimensional facial surface (FastSCAN Cobra™, Polhemus, Applied Research Associates NZ Scanning Ltd., Christchurch, New Zealand) of each participant was scanned using six reference landmarks (the external meatus of each ear, three points on the forehead, and the nasion) to superimpose each MEG datum on individual magnetic resonance imaging (MRI) datum.

Cortical responses to visual stimuli were measured with the participant lying in a comfortable supine position on a bed in a magnetically shielded room, using a whole-head 160-channel MEG system equipped with superconducting quantum interference device gradiometers (PQ1160C, Yokogawa Electric Corporation, Kanazawa, Japan). Before and after data acquisition, the positions of five head marker coils (two at the external meatus of each ear and three points on the forehead) were obtained to estimate each participant’s head position against the MEG sensors. The MEG data were recorded at a sampling rate of 1000 Hz, with an online low-pass filter at 200 Hz. Prior to the MEG recording, each participant was instructed to relax and look at the monitor without moving the head or body during the measurement, and to press the button placed under the right hand when the picture of a red circle was displayed. Throughout the measurement, the state of each participant was observed using a video camera.

Individual anatomical MRI datum was acquired using a 3.0-T whole-body magnetic resonance scanner equipped with a 24-channel-head coil (3-T Discovery MR 750w system, GE Healthcare, Milwaukee, WI, USA). A three-dimensional silent T1-weighted sagittal protocol was used, with the following imaging parameters: repetition time/echo time = 880/0.016 ms, field of view = 240 mm, matrix = 240 × 240, slice thickness = 1.0 mm, 0.5-mm gap, number of slices = 480, and acquisition time = 5 min 10 s.

### Data analysis

The epochs extended from 100 ms before stimulus onset to 1000 ms after stimulus onset. The data obtained 100 ms before the stimulus onset was used as the baseline.

#### Visual evoked fields

**Fig. 2:**
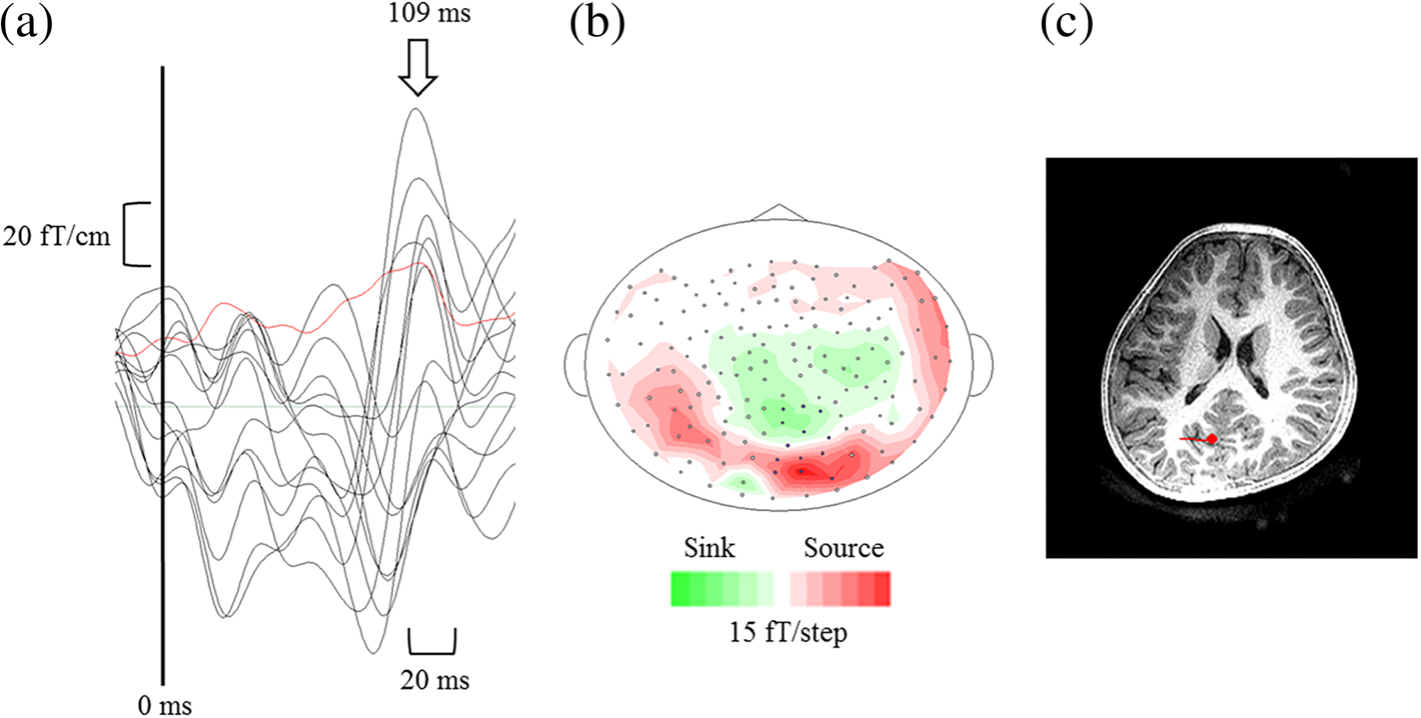
Representative figures of visual evoked fields (VEFs). The VEFs for the Original image in a child with TD have been shown. **a** VEF waveforms from the 13–15 sensors in the primary visual cortex. Root mean square values have been presented by a red waveform. The arrow represents the M100 peak. The *X*-axis indicates the latency in ms. The *Y*-axis indicates the amplitude in fT. **b** Isomagnetic field maps at the M100 peak. **c** Dipole sources overlaid on the individual magnetic resonance images of the participants. Typically developing (TD)

Visual evoked fields were determined using MEG Laboratory software (Yokogawa Electric Corporation). We determined the visual M100 peak, reflecting object-sensitive activity [40]. The epochs contaminated by artifacts such as head movements, eye blinks, and muscular activity were removed manually following visual inspection of the MEG signals. The remaining epochs were averaged for each image and participant using a high-pass filter with a cut-off frequency of 3 Hz, and low-pass filtered with a cut-off frequency of 40 Hz [26, 28]. Root mean square values were calculated using 13–15 sensors in the primary visual cortex, and the M100 peak latency was specified by the time window from 85 to 135 ms. Furthermore, for each peak, we confirmed that the estimated equivalent current dipole showed a goodness of fit of over 80% (Fig. 2).

#### Cortical activation

Cortical activation in response to the images was examined using Brainstorm software, which is freely available for download online under the GNU general public license (http://neuroimage.usc.edu/brainstorm) [41]. Each individual cortical surface was created from the MRI data using FreeSurfer 5.3.0 image analysis software (http://surfer.nmr.mgh.harvard.edu/) [42]. A band-pass filter from 1 to 40 Hz was applied to the MEG signals. The epochs exceeding 1500 fT/cm were rejected. The artifacts derived from heartbeats and eye movements were also eliminated using signal space projections [41]. All epochs, except for the removed ones, were averaged and transformed into a z-score for each type of image for each participant.

The overlapping sphere model was used to compute the head model individually [41, 43]. Source estimation was performed using weighted minimum-norm estimation, adapted from depth-weighted minimum linear L2 norm estimators [41, 44]. These functional images obtained from the MEG signals were projected onto the Colin 27 brain template, and used to create the grand-average images for all the groups.

**Fig. 3:**
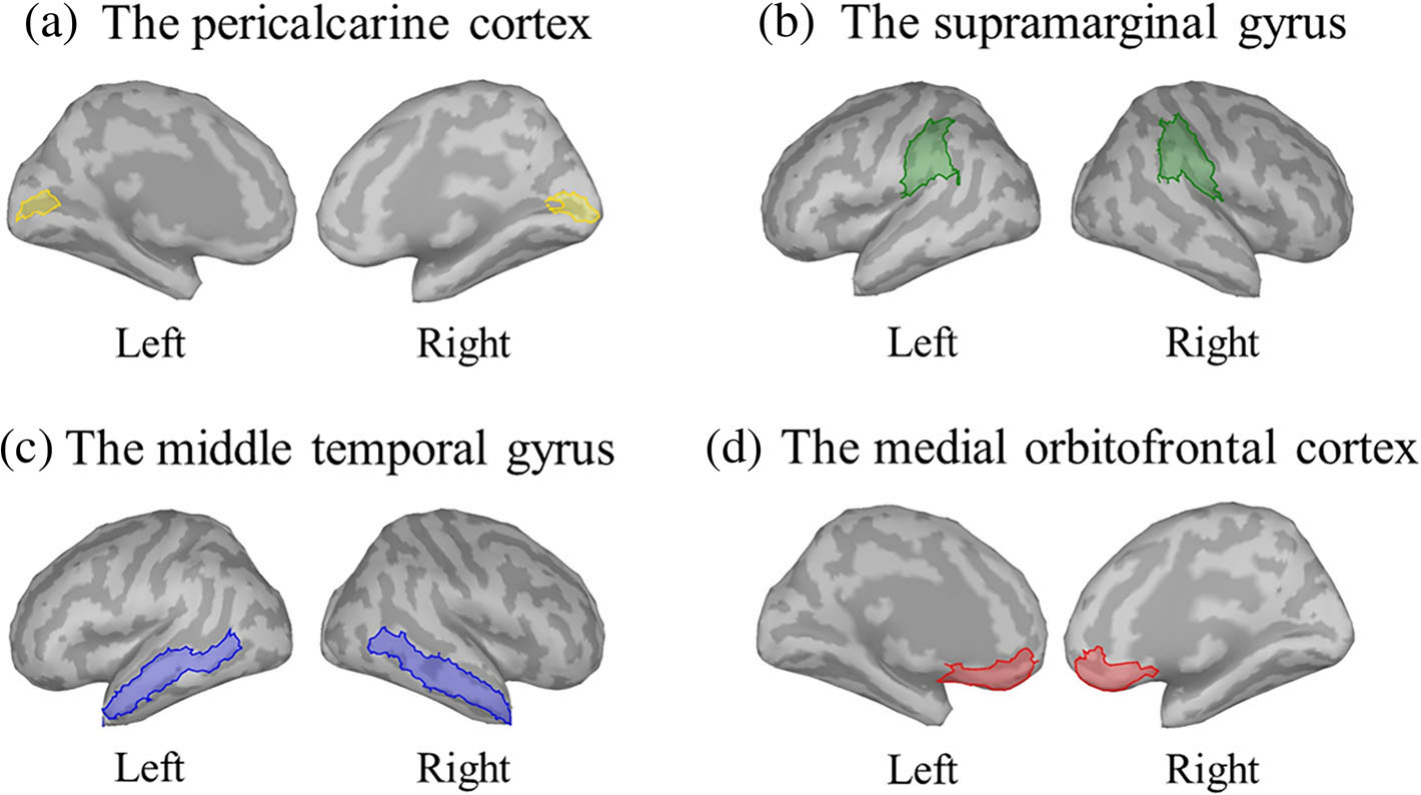
Regions of interest. Regions of interest, including the **a** bilateral pericalcarine cortex (PCAL), **b** supramarginal gyrus (SMG), **c** middle temporal gyrus (MTG), and **d** medial orbitofrontal cortex (mOFC), were determined with the Desikan-Killiany atlas

Regions of interest (ROIs) were determined with the Desikan-Killiany atlas covering event-related extensive activities [45], and the increasing cortical activation for both groups was confirmed by visual inspection. The time course of cortical activation in each ROI was based on visual inspection of the prominent peak activity in each individual’s averaged data, and identified between 100 and 200 ms in the pericalcarine cortex (PCAL), 180 and 380 ms in the supramarginal gyrus (SMG), 200 and 400 ms in the middle temporal gyrus (MTG), and 350 and 500 ms in the medial orbitofrontal cortex (mOFC) (Fig. 3). Activated intensities were determined by averaging the maximum intensities in each ROI across the time course.

#### Ratio of neural responses

We calculated the ratio of neural responses, such as latency and activated intensity, to be able to clearly identify the differences in the responses to bright images based on the individual response to the Original image. This is because changes in individual neural response to the types of images may be canceled out by individual differences in the analysis using the difference score [46].

#### Statistical analysis

A chi-squared test was used to compare the sex ratio between groups. Analysis of variance was used to compare the demographic variables such as age, FSIQ, SP visual score, and CBCL total score. We performed analysis of covariance with group (ASD/TD) as a between-subject factor, and participants’ age and FSIQ as covariates, to determine the M100 latencies in responses to the Original image and the M100 latency ratios.

Activated intensities of the responses to the Original image and the activated intensity ratios were assessed using repeated measure analysis of covariance, with the hemisphere (left/right) as a within-subject factor, group (ASD/TD) as a between-subject factor, and participants’ age and FSIQ as covariates. If the results of Mauchly’s sphericity test were significant, the Greenhouse-Geisser correction was applied to the data. Because eight locations were investigated (four ROIs in each hemisphere), Bonferroni’s correction was applied to the multiple comparison analyses (*p <* 0.006). Pearson’s correlation analyses were performed for the following pairs: SP visual item scores and CBCL total scores, SP visual item scores and the activated intensities, and SP visual item scores and the activated intensity ratios. All statistical analyses were performed using SPSS version 24.0 (IBM Corp., Tokyo, Japan).

## Results

### Demographics

**Fig. 4:**
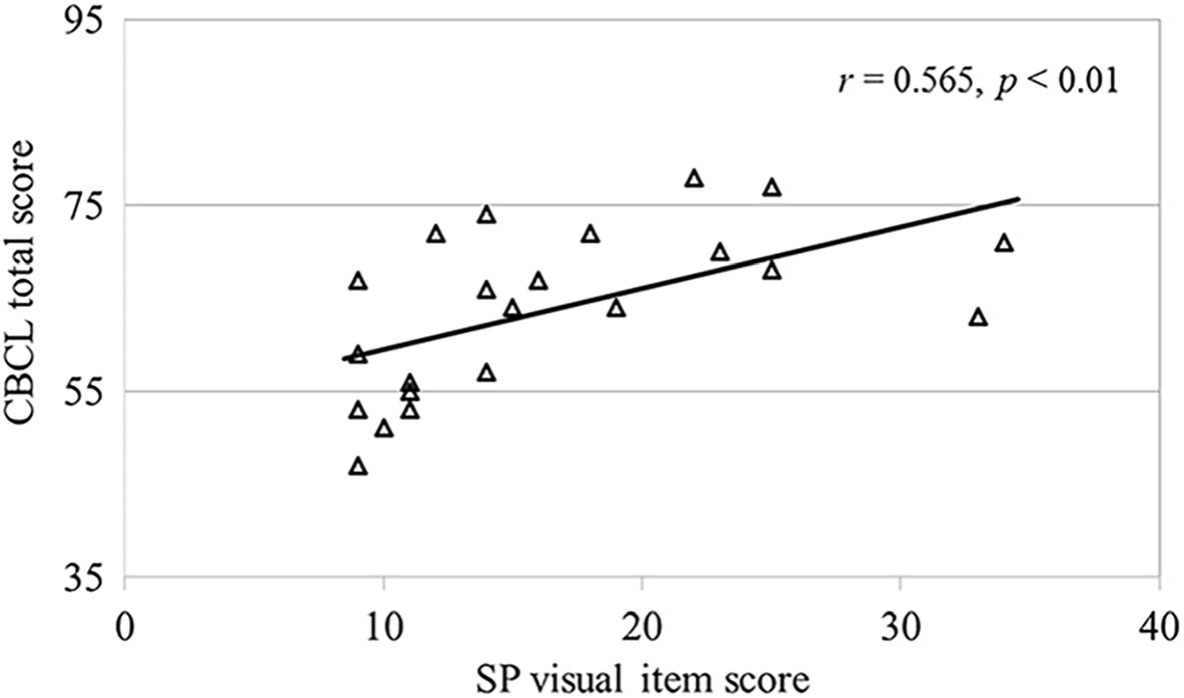
Correlation between the SP visual score and CBCL total score for the ASD group. The severity of visual abnormalities was associated with the seriousness of behavioral problems (*p* < 0.01). Sensory profile (SP); Child Behavior Checklist (CBCL); autism spectrum disorder (ASD)

There was no significant inter-group difference in the sex ratio (ASD, 21:1; TD, 21:2; *χ*^*2*^ = 0.31; *p* = 0.577), age (ASD, 10.95 ± 2.01 years; TD, 10.13 ± 2.80 years; *F* (1, 43) = 1.28; *p* = 0.265), and FSIQ (ASD, 102.05 ± 15.83; TD, 107.70 ± 10.85; *F* (1, 43) = 1.97; *p* = 0.168) (Table 1). However, the visual item scores of SP were significantly higher in the ASD group, which indicated more abnormal sensitivity (ASD, 16.50 ± 7.57; TD, 10.48 ± 1.97; *F* (1, 43) = 13.60; *p* < 0.01; *η*_*p*_^*2*^ = 0.24). Moreover, the total CBCL scores were higher for the ASD group, which indicated more problematic behavior (ASD, 63.82 ± 8.82; TD, 50.17 ± 7.83; *F* (1, 43) = 30.16; *p* < 0.001; *η*_*p*_^*2*^ = 0.41). Furthermore, the SP visual item scores were significantly correlated with the CBCL total scores in the ASD group (*r* = 0.565, *p* < 0.01; Fig. 4). In addition, this statistical significance and strength of the correlation remained even after controlling for the participants’ age and FSIQ (*r* = 0.506, *p* < 0.05).

**Table 1:** Characteristics of the study participants

Group	TD children	Children with ASD	χ^2^
N (male:female)	23 (21:2)	22 (21:1)	0.31
	Mean ± SD	Mean ± SD	*F*
Age (years)	10.13 ± 2.80	10.95 ± 2.01	1.28
FSIQ	107.70 ± 10.85	102.05 ± 15.83	1.97
SP visual item scores	10.48 ± 1.97	16.50 ± 7.57	13.60**
CBCL total scores	50.17 ± 7.83	63.82 ± 8.82	30.16***

Typically developing (TD); autism spectrum disorder (ASD); standard deviation (SD); full-scale intelligence quotient of Wechsler Intelligence Scale for Children, Fourth Edition (FSIQ); Sensory Profile (SP); Child Behavior Checklist (CBCL). **p < 0.01; ***p < 0.001

### M100 latencies

#### Comparison of M100 latencies in response to the Original, Dot noise, and Blind images between the two groups

We examined the mean M100 latencies in response to the Original image at the primary visual cortex. The mean M100 latencies in response to the Original image in the ASD and TD groups were identified at 108.95 ± 10.50 and 108.78 ± 11.71 ms, respectively. There was no significant inter-group difference (*F* (1, 41) = 0.06, *p* = 0.797).

Furthermore, we examined the ratios of the M100 latencies in response to the Dot noise and Blind images to that in response to the Original image. Both M100 latencies ratios did not show significant inter-group differences (Dot noise/Original: ASD, 1.02 ± 0.09; TD, 1.05 ± 0.14; *F* (1, 41) = 0.13; *p* = 0.725; Blind/Original: ASD, 1.00 ± 0.11; TD, 1.01 ± 0.13; *F* (1, 41) = 0.03; *p* = 0.872).

### Comparison of grand-averaged activated intensities between the two groups

**Fig. 5:**
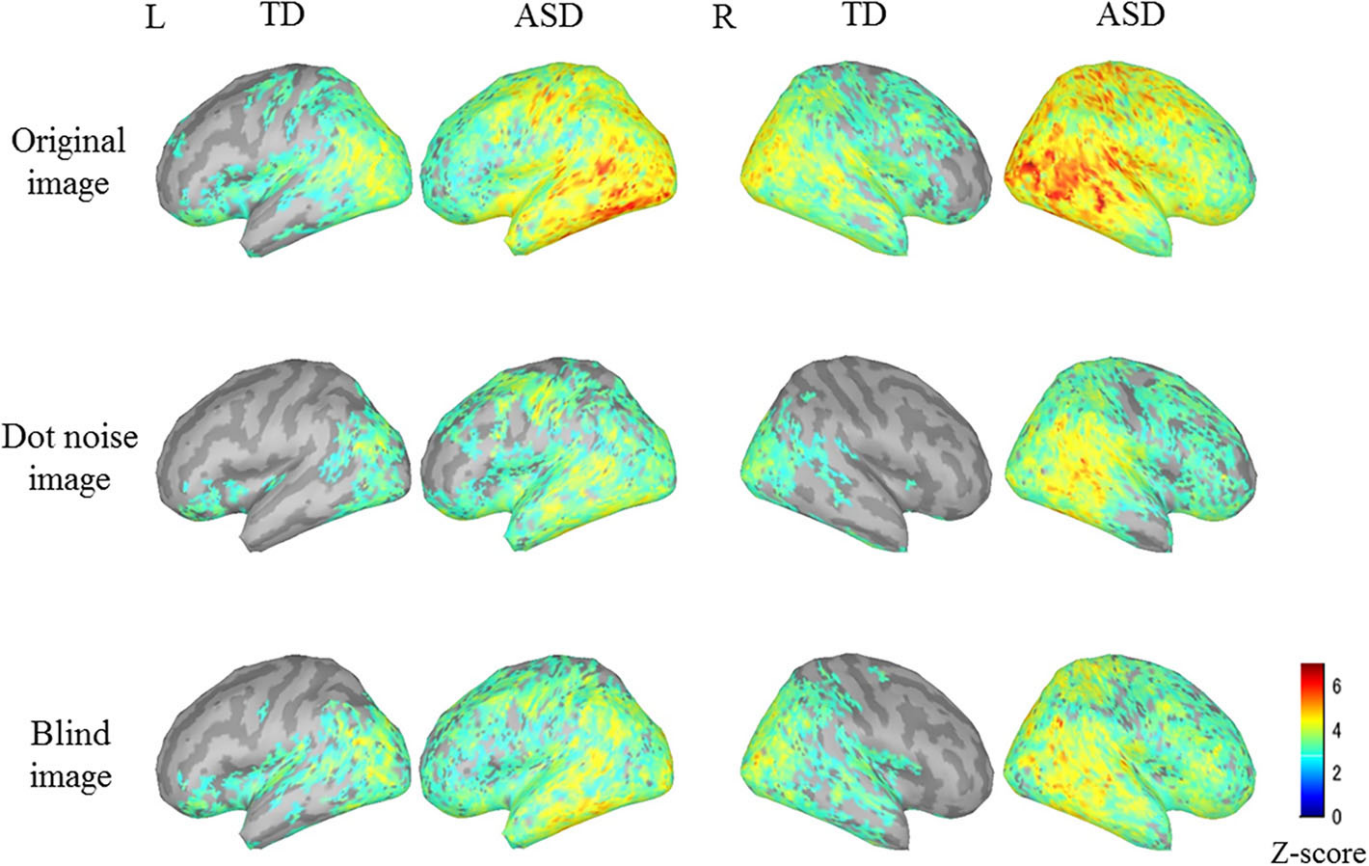
Grand-average cortical activation in response to each image for each group. The mean cortical activation from 200 to 400 ms after stimulus onset are indicated on the normalized brain surface for each group. The activation patterns at the occipital, temporal, and parietal cortices in the TD group have been shown. These activations were decreased in the case of bright (Dot noise and Blind) images compared with the Original image. The cortical activation in response to the Original image increased in the ASD group compared with that in the TD group. The attenuation of the response to bright image in the ASD group weakened compared with that in the TD group. Typically developing (TD); autism spectrum disorder (ASD)

The mean grand-averaged cortical activation from 200 to 400 ms was visually assessed, as shown in Fig. 5. Activations were observed at the occipital, temporal, and parietal cortices in the TD group. These activations were decreased in the case of the Dot noise and Blind images compared with the Original image. The cortical activation in response to the Original image in the ASD group increased compared with that in the TD group. In addition, the attenuation due to the Dot noise and Blind images was weaker in the ASD group than that in the TD group.

### Activated intensities in the ROIs

#### Comparison of activated intensities in response to the Original image between the two groups

**Fig. 6:**
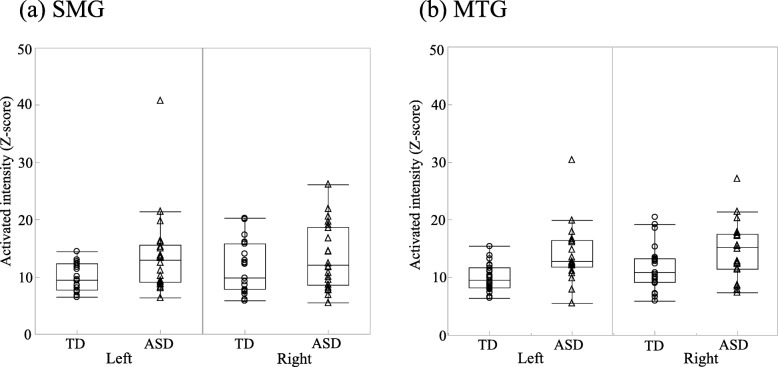
Activated intensities in response to the Original image for each group. **a** Activated intensities in response to the Original image in the left SMG between 180 and 380 ms were increased in the ASD group compared with the TD group (*p* < 0.05). **b** Activated intensities in response to the Original image in the left MTG between 200 and 400 ms were significantly increased in the ASD group compared with the TD group (Left: *p* < 0.006, Right: *p* < 0.05). Supramarginal gyrus (SMG); autism spectrum disorder (ASD); typically developing (TD); middle temporal gyrus (MTG)

We examined the activated intensities in response to the Original image for each ROI. The mean activated intensities in response to the Original image in the PCAL showed no significant interaction between group and hemisphere (ASD: L 13.79 ± 5.80, R 12.77 ± 5.40; TD: L 11.29 ± 8.66, R 12.03 ± 7.76; *F* (1, 41) = 0.30; *p* = 0.586) or main effect of group (*F* (1, 41) = 1.18, *p* = 0.283). The mean activated intensities in the SMG showed a marginally significant main effect of group (ASD: L 13.71 ± 7.18, R 13.51 ± 5.60; TD: L 9.95 ± 2.31, R 11.60 ± 4.36; *F* (1, 41) = 3.36; *p* = 0.074; *η*_*p*_^*2*^ = 0.08; Fig. 6a), which showed that the response of the ASD group was enhanced to a significantly greater extent in the left hemisphere than that of the TD group (*p* < 0.05; *η*_*p*_^*2*^ = 0.10), without any inter-group differences in the right hemisphere (*p* > 0.346). However, the difference did not remain after correcting for multiple comparisons. Furthermore, the mean activated intensities in the MTG showed a significant main effect of group (ASD: L 14.09 ± 4.94, R 14.74 ± 4.81; TD: L 9.98 ± 2.33, R 11.77 ± 3.90; *F* (1, 41) = 11.34; *p* < 0.01; *η*_*p*_^*2*^ = 0.22; Fig. 6b). This showed that although the response of the ASD group was enhanced to a significantly greater extent in the bilateral MTG than that of the TD group (*p* < 0.05; *η*_*p*_^*2*^ > 0.10), only the difference in the left hemisphere remained after correcting for multiple comparisons. There was also a trend of an interaction in the mOFC (ASD: L 10.43 ± 5.81, R 9.76 ± 2.93; TD: L 9.07 ± 5.39, R 10.37 ± 5.98; *F* (1, 41) = 3.22; *p* = 0.080; *η*_*p*_^*2*^ = 0.07), whereas there was no significant inter-group difference in any hemisphere (*p* > 0.414).

In addition, we performed correlation analysis to reveal the relationship between the mean activated intensities showing inter-group difference and the visual abnormalities. No significant correlation between the mean activated intensities in the left SMG and the SP visual item scores was observed in the ASD group (*r* = − 0.171, *p* > 0.447); similarly, no significant correlation was observed in the bilateral MTG (L: *r* = − 0.003, *p* > 0.989, R: *r* = − 0.259, *p* > 0.245).

#### Comparison of attenuation ratios of the activated intensities in response to the bright images between the two groups

Furthermore, we explored the ratios of the activated intensities in response to the Dot noise and Blind images to that in response to the Original image, for each ROI.

Concerning the PCAL, both the activated intensity ratios showed no interaction (Dot noise/Original ASD: L 0.85 ± 0.35, R 0.98 ± 0.43; TD: L 1.08 ± 0.51, R 1.09 ± 0.73; *F* (1, 41) = 0.46; *p* = 0.503; Blind/Original ASD: L 0.83 ± 0.41, R 0.93 ± 0.35; TD: L 0.81 ± 0.36, R 0.91 ± 0.42; *F* (1, 41) = 0.02; *p* = 0.884) or main effect of group (Dot noise/Original: *F* (1, 41) = 1.67, *p* = 0.203; Blind/Original: *F* (1, 41) = 0.01, *p* = 0.931).

**Fig. 7:**
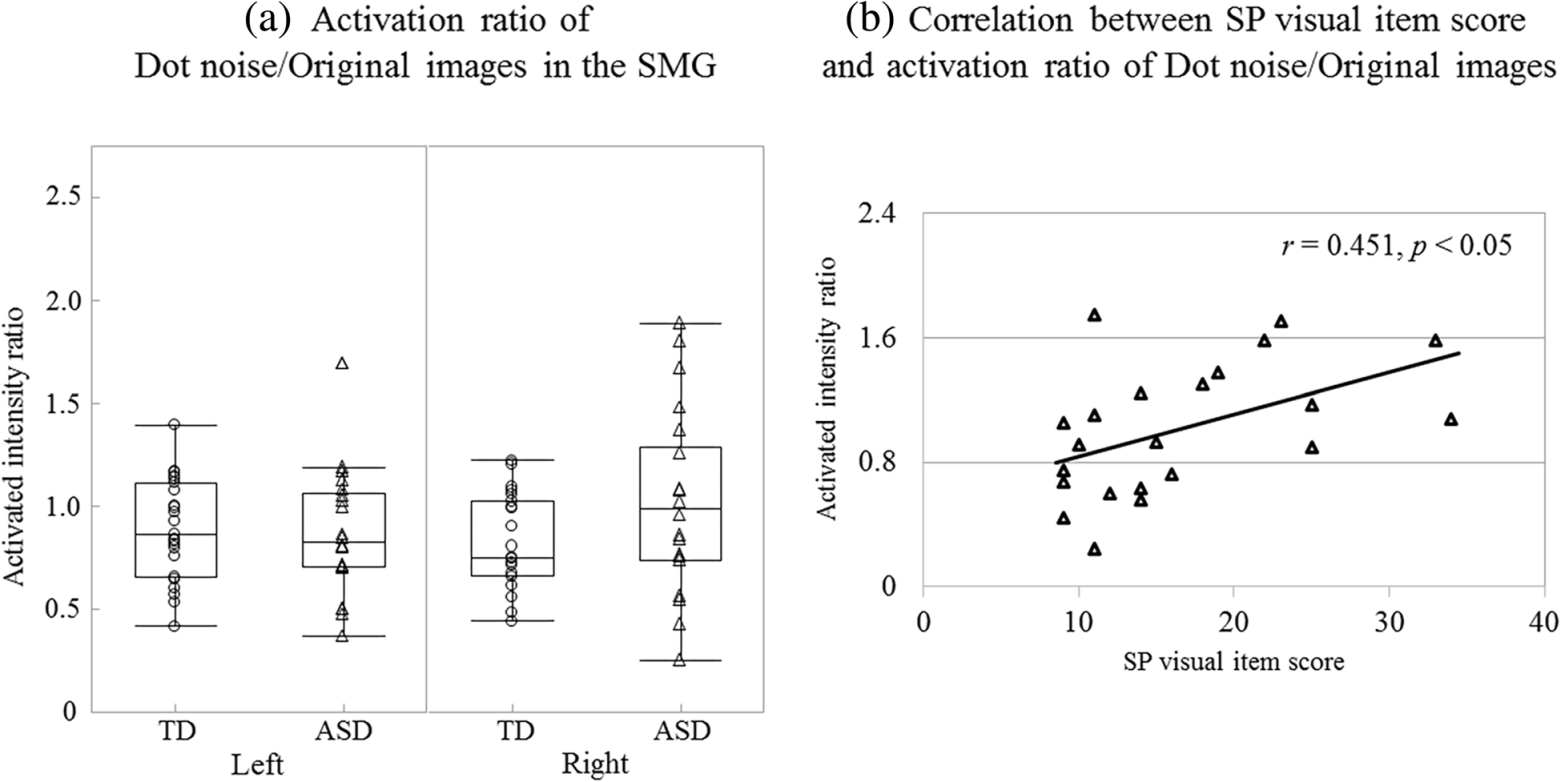
Attenuation of the response to the Dot noise image and abnormal visual sensitivity. **a** In the right SMG, the activation ratio of Dot noise/Original images between 180 and 380 ms was increased in the ASD group compared with the TD group (*p* < 0.05). This indicates that the attenuation of the response to the Dot noise image in the ASD group was weaker. **b** Correlation between the SP visual item score and activation ratio for the ASD group. The patients with ASD exhibiting more severe visual abnormalities showed greater activated intensities in response to the Dot noise image (*p* < 0.05). Supramarginal gyrus (SMG); autism spectrum disorder (ASD); typically developing (TD); Sensory Profile (SP)

The mean Dot noise/Original ratio in the SMG showed significant interaction (ASD: L 0.86 ± 0.30, R 1.01 ± 0.43; TD: L 0.89 ± 0.25, R 0.82 ± 0.23; *F* (1, 41) = 5.41; *p* < 0.05; *η*_*p*_^*2*^ = 0.12; Fig. 7a), which indicated that the response of the ASD group increased significantly in the right hemisphere compared with that of the TD group (*p* < 0.05; *η*_*p*_^*2*^ = 0.09); however, no differences were observed in the left hemisphere (*p* > 0.510). This difference did not remain after correcting for multiple comparisons. Similar results were obtained for the Blind/Original ratio (ASD: L 0.99 ± 0.49, R 1.05 ± 0.42; TD: L 1.07 ± 0.42, R 0.89 ± 0.30; *F* (1, 41) = 6.01; *p* < 0.05; *η*_*p*_^*2*^ = 0.13; Additional file 1a), which revealed that the ASD group showed a trend of lesser attenuation of the response to the Blind image in the right hemisphere than the TD group (*p* = 0.079; *η*_*p*_^*2*^ = 0.07).

The mean Dot noise/Original ratio in the MTG showed a trend of an interaction (ASD: L 0.86 ± 0.28, R 1.07 ± 0.50; TD: L 1.03 ± 0.33, R 0.93 ± 0.35; *F* (1, 41) = 4.05; *p* = 0.051; *η*_*p*_^*2*^ = 0.09), which showed that the response of the ASD group was significantly reduced in the left hemisphere compared with that of the TD group (*p* < 0.05; *η*_*p*_^*2*^ = 0.11). The difference remained after correcting for multiple comparisons. In addition, the mean Blind/Original ratio in the MTG showed no interaction (ASD: L 0.86 ± 0.42, R 0.97 ± 0.46; TD: L 1.08 ± 0.43, R 0.97 ± 0.29; *F* (1, 41) = 1.28; *p* = 0.265) or main effect of group (*F* (1, 41) = 2.50, *p* = 0.122).

Concerning the mOFC, the mean Dot noise/Original ratio showed no interaction (ASD: L 1.00 ± 0.52, R 0.88 ± 0.37; TD: L 1.04 ± 0.51, R 1.04 ± 0.66; *F* (1, 41) = 1.13; *p* = 0.295) or main effect of group (*F* (1, 41) = 1.05, *p* = 0.311). The mean Blind/Original ratio showed significant interaction (ASD: L 1.02 ± 0.49, R 1.02 ± 0.51; TD: L 1.17 ± 0.77, R 0.96 ± 0.52; *F* (1, 41) = 5.02; *p* < 0.05; *η*_*p*_^*2*^ = 0.11), whereas there was no significant inter-group difference in any hemisphere (*p* > 0.316).

In addition, we performed correlation analysis to examine the relationship between the activation ratios showing the inter-group difference and the visual abnormalities.

Regarding the right SMG, each mean activated intensity ratio was significantly correlated with SP visual item scores in the ASD group (Dot noise/Original: *r* = 0.451, *p* < 0.05, Fig. 7b; Blind/Original: *r* = 0.485, *p* < 0.05; Additional file 1b). The correlation remained significant even after controlling for the participants’ age and FSIQ (Dot noise/Original: *r* = 0.464, *p* < 0.05; Blind/Original: *r* = 0.522, *p* < 0.05).

Regarding the left MTG, no significant correlation between the mean Dot noise/Original ratio and SP visual item scores was observed in the ASD group (*r* = − 0.050, *p* > 0.825).

## Discussion

This study demonstrated that children with ASD showed increased cortical activation in response to the Original image in the left SMG and MTG but not in the primary visual cortex and lesser attenuation to the bright images compared with TD children. In addition, these attenuation ratios were associated with the severity of visual abnormalities in the right SMG.

A previous study demonstrated a similar association between auditory abnormalities and behavioral problems [28]. The individuals with ASD often complain of abnormal symptoms of the multisensory systems. Therefore, these abnormal symptoms, independent of the specific sensory system, may have common features in the sensory network. Further, such symptoms may lead to difficulty of the patients to adapt in their daily lives and cause their behavioral problems.

This study showed increased cortical activation in response to the Original image in the left SMG and bilateral MTG in the ASD group compared with the TD group. Previous studies have indicated that the left inferior parietal cortex, including the SMG, is involved in object-based attention and object usage [47, 48]. In addition, the temporal cortex, including the MTG, plays an important role in object perception and recognition [49].

Concerning the temporal cortex in particular, Hutsler and Zhang [50] have reported greater dendritic spine densities in layer II and V of the temporal cortex in patients with ASD. In addition, a previous auditory study has demonstrated both reduced relative γ-aminobutyric acid-ergic concentration and gamma-band response deficits in response to auditory stimuli in the temporal cortex in the ASD group [51]. Therefore, the enhanced responses to visual stimulus in ASD might possibly be caused by alterations in excitability in these areas.

Regarding latencies in the occipital cortex in the early stage of visual pathway, the ASD does not show any differences compared with the TD group in the P100 latencies to the pattern-reversal stimuli [52, 53]. In the present study, the M100 latencies in response to the Original image and the M100 ratios for the different types of images also did not show any differences in the occipital cortex between the two groups. However, according to previous auditory studies, prolonged latencies in the primary auditory cortex showed significant association with the severity of abnormal auditory sensitivity, which was concluded to involve the delayed or atypical myelination in children with ASD [28, 54, 55]. The pattern of these differences might be reflected in the sequence of myelination [56], that is, myelination of the optic radiation is supposed to occur earlier than that of the acoustic radiation. Therefore, we speculate that myelination of the visual cortical region might be less likely to contribute to the visual abnormalities in ASD.

Concerning the dependence of the differences in activated intensities on the properties of images, previous studies have reported that the enhanced luminance stimuli induce a higher response amplitude in the occipital cortex [57, 58]. However, in the present study, activated intensities did not differ according to the types of images in the TD group (Additional file 2). These inconsistent results might depend on the degree of luminance. Although assessments in the previous studies were performed under relatively extreme conditions, such as 180 cd/m^2^ and 11 cd/m^2^, or 55 cd/m^2^ and 0.76 cd/m^2^, respectively [57, 58], luminance of the images used in the present study showed small differences, with the maximum difference being between 85 cd/m^2^ and 48 cd/m^2^. As patients with ASD report not perceiving “brightness” in response to high-luminance images, but “sparkling sensation” in response to the normal-luminance images, we believe that the images selected in the present study could simulate their experiences. Therefore, the differences in the contrast of the images could result in inconsistent findings associated with the adaptation systems, such as contraction of pupil and gating system of the thalamus or neural habituation system. On the other hand, a previous study has revealed that the effect of luminance was stronger in the parieto-occipital region [59]. Indeed, inter-group differences in the response to bright images were prominent in the parietal region in our study. Future studies are needed to examine the mechanism underlying these differences.

The change in the response to the bright images in the right SMG, which is associated with visual abnormalities, were less attenuated in the ASD group in the late stage. These attenuation ratios are considered to reflect the degree of neural suppression of the response to bright images. Previous studies have revealed that the temporoparietal junction (TPJ), including the SMG, was composed of the ventral attentional network, which indicated attention specialized for the detection of behaviorally relevant stimuli, and was largely lateralized to the right hemisphere [18]. In addition, children with ASD show hyper-connectivity of the ventral attentional network and thalamocortical circuit in the right TPJ compared with TD children [60, 61]. Therefore, the hyper-connectivity between these regions, in addition to the hyperexcitability of the local area of the right TPJ, may contribute to the neural mechanism underlying visual abnormalities in ASD. Furthermore, since the right TPJ has been shown to preferentially respond to behaviorally relevant stimuli across sensory modalities, such as visual, auditory, or tactile modalities [62, 63], the general neural basis of the sensory impairment in ASD may involve atypical stimulus-driven attention in the late stage. On the other hand, the right TPJ plays a key role in social cognition [64], which is crucially influenced in the aforementioned attentional mechanism [65]. Especially, social impairment is a core characteristic feature of ASD, and studies have reported abnormalities in the right TPJ during theory of mind tasks [66, 67]. In addition, structural imaging studies have revealed that the right TPJ shows earlier gray matter maturation in ASD [68, 69]. These structural and functional alterations in the right TPJ may better explain the symptomatology of ASD.

In addition, the present study showed no difference in the activated intensity ratios in the mOFC in the late stage between the two groups. The OFC is involved in emotional regulation, and patients with OFC lesions fail to habituate to mildly aversive stimulus [70, 71]. Children with ASD often show difficulty in emotional and behavioral regulation in response to visual stimuli in their daily lives. Therefore, the visual stimuli used in this study might be less emotionally relevant than those used in previous studies.

Reconsidering our results in the light of enhanced visual perception in ASD revealed that the affected children might be able to better detect objects in bright environments. This implies that visual sensory abnormality is inextricably linked to enhanced visual perception in ASD based on the demand of the situation [12, 72].

There are several limitations to this study. Firstly, the size of the study population was relatively small. Future studies with larger study population and narrower age range are needed to better characterize the inter-group differences, which may provide more robust evidence for the neural bases. Secondly, some of the inter-group differences reported in this study did not remain after correcting for multiple comparisons. This may probably be due to the small size of the study population, or possibly a type 1 error. Therefore, these results should be interpreted with caution. Thirdly, the present study population exhibited a lack of sex-related differences. Although it is difficult to recruit girls with ASD considering the skewed sex ratio of high-functioning ASD [73], we did not examine any sex-related differences. As women with high-functioning autism spectrum conditions also show more lifetime sensory symptoms than men with this condition [74], future sensory studies are also needed to focus on the sex-related differences in ASD. Lastly, the present study lacks a longitudinal design. Future studies with a longitudinal design would allow examination of the changes in visual symptoms and neural activation throughout the children’s development, and may provide clearer evidence of the sensory abnormalities in ASD. This may facilitate adequate intervention depending on the developmental stage of the patient.

## Conclusions

The present MEG study revealed that children with ASD in the late stage show lesser attenuation than TD children of the response to the bright images compared with the Original image, and their attenuation ratios are associated with the severity of visual abnormalities in the right SMG. We believe that attenuated stimulus-driven neural suppression contributes to the visual abnormality in ASD. Our findings provide better understanding of the visual abnormality in ASD, and may lead to more effective diagnosis and earlier intervention.

## Additional files

Additional file 1: Attenuation of the response to the Blind image and abnormal visual sensitivity. (a) In the right SMG, the activation ratio of Blind/Original images between 180 and 380 ms was increased in the ASD group compared with the TD group (*p* < 0.10). This indicates that the attenuation of the response to the Blind image in the ASD group was weaker. (b) Correlation between the SP visual item score and activation ratio for the ASD group. The patients with ASD exhibiting more severe visual abnormalities showed greater activated intensities in response to the Blind image (*p* < 0.05). supramarginal gyrus (SMG); autism spectrum disorder (ASD); typically developing (TD); Sensory Profile (SP) (TIF 255 kb)

Additional file 2: Activated intensities in the PCAL in the TD group in response to different types of images. We tested the difference in activated intensities in the PCAL in the TD group in response to the different types of images. The mean activated intensities showed no significant interaction (Original image: L 11.29 ± 8.66, R 12.03 ± 7.76; Dot noise image: L 9.92 ± 3.62, R 11.15 ± 7.17; Blind image: L 7.41 ± 2.85, R 9.12 ± 3.32; *F* (1.393, 27.854) = 0.62; *p* = 0.490) or main effect of stimulus (*F* (1.482, 29.631) = 0.62, *p* = 0.496). pericalcarine cortex (PCAL); typically developing (TD) (DOCX 15 kb)

## Abbreviations

ASD: Autism spectrum disorder
CBCL: Child Behavior Checklist
DSM-5: Diagnostic and Statistical Manual of Mental Disorders, Fifth Edition
FSIQ: Full-scale intelligence quotient
MEG: Magnetoencephalography
mOFC: medial orbitofrontal cortex
MRI: Magnetic resonance imaging
MTG: Middle temporal gyrus
PCAL: Pericalcarine cortex
ROI: Region of interest
SCQ: Social Communication Questionnaire
SMG: Supramarginal gyrus
SP: Sensory profile
TD: Typically developing
TPJ: Temporoparietal junction
