# Supplementary figures and images for: Lesser suppression of response to bright visual stimuli and visual abnormality in children with autism spectrum disorder: a magnetoencephalographic study
Source: J Neurodev Disord. 2019 Jun 14;11:9. doi: 10.1186/s11689-019-9266-0 (PMC6570891; doi:10.1186/s11689-019-9266-0)

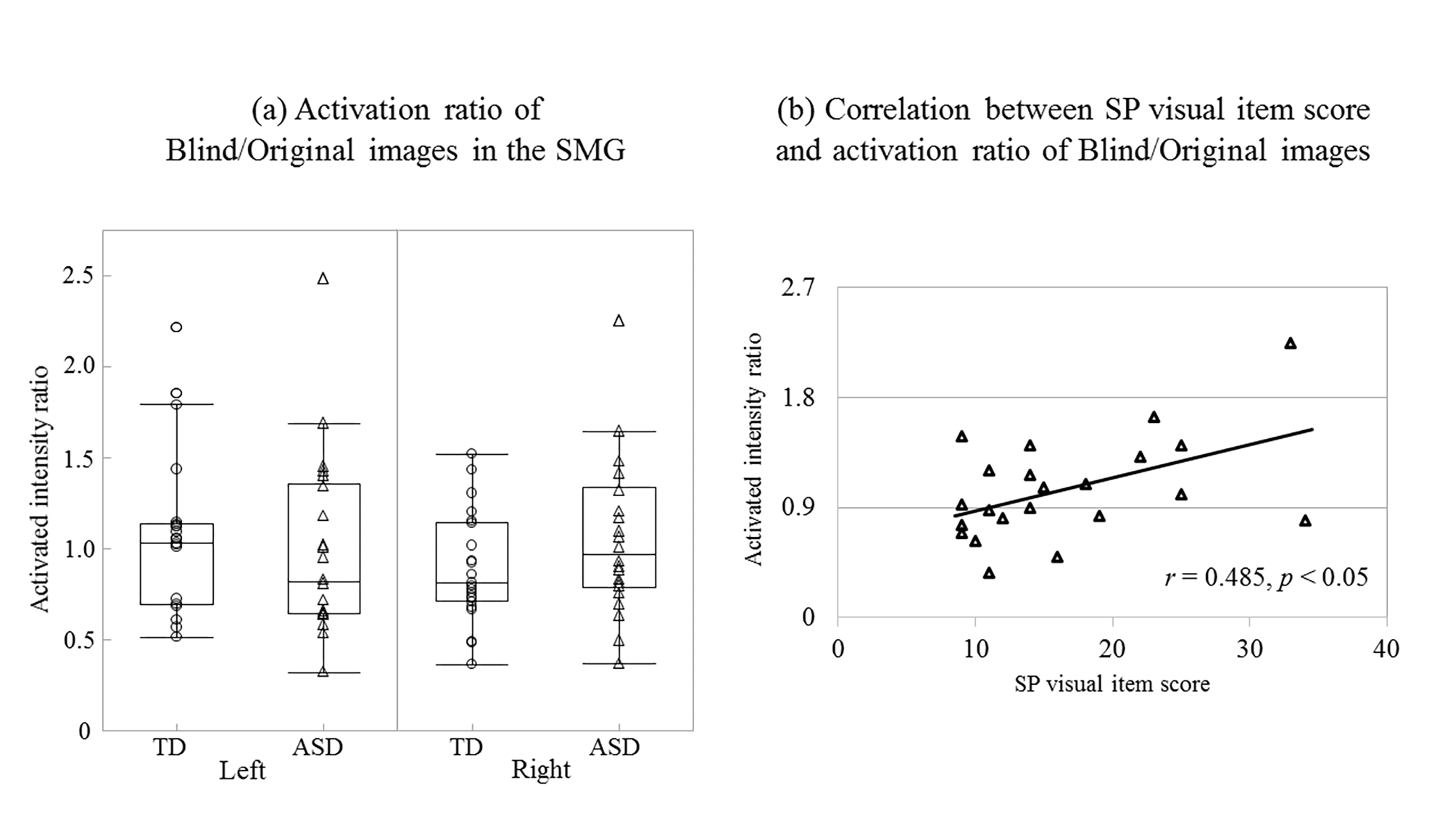

Supplement: Supplementary file 1 — Attenuation of the response to the Blind image and abnormal visual sensitivity. (a) In the right SMG, the activation ratio of Blind/Original images between 180 and 380 ms was increased in the ASD group compared with the TD group (p < 0.10). This indicates that the attenuation of the response to the Blind image in the ASD group was weaker. (b) Correlation between the SP visual item score and activation ratio for the ASD group. The patients with ASD exhibiting more severe visual abnormalities showed greater activated intensities in response to the Blind image (p < 0.05). supramarginal gyrus (SMG); autism spectrum disorder (ASD); typically developing (TD); Sensory Profile (SP) (TIF 255 kb) [file 11689_2019_9266_MOESM1_ESM.tif]
