# Supplementary material for: Lesser suppression of response to bright visual stimuli and visual abnormality in children with autism spectrum disorder: a magnetoencephalographic study
Source: J Neurodev Disord. 2019 Jun 14;11:9. doi: 10.1186/s11689-019-9266-0 (PMC6570891; doi:10.1186/s11689-019-9266-0)
Supplement: Supplementary file 2 — Activated intensities in the PCAL in the TD group in response to different types of images. We tested the difference in activated intensities in the PCAL in the TD group in response to the different types of images. The mean activated intensities showed no significant interaction (Original image: L 11.29 ± 8.66, R 12.03 ± 7.76; Dot noise image: L 9.92 ± 3.62, R 11.15 ± 7.17; Blind image: L 7.41 ± 2.85, R 9.12 ± 3.32; F (1.393, 27.854) = 0.62; p = 0.490) or main effect of stimulus (F (1.482, 29.631) = 0.62, p = 0.496). pericalcarine cortex (PCAL); typically developing (TD) (DOCX 15 kb) [file 11689_2019_9266_MOESM2_ESM.docx]

We tested the difference in activated intensities in the PCAL in the TD group in response to the different types of images. The mean activated intensities showed no significant interaction (Original image: L 11.29 ± 8.66, R 12.03 ± 7.76; Dot noise image: L 9.92 ± 3.62, R 11.15 ± 7.17; Blind image: L 7.41 ± 2.85, R 9.12 ± 3.32; *F* (1.393, 27.854) = 0.62; *p* = 0.490) or main effect of stimulus (*F* (1.482, 29.631) = 0.62, *p* = 0.496). pericalcarine cortex (PCAL); typically developing (TD)
